# Granulomatosis with Polyangiitis Presenting as a Choroidal Tumor

**DOI:** 10.1155/2015/271823

**Published:** 2015-04-08

**Authors:** Taro Masuda, Yasumori Izumi, Hayato Takeshita, Chieko Kawahara, Yoshika Tsuji, Hirokazu Kurohama, Nozomi Iwanaga, Miwako Inamoto, Keiichi Kase, Masahiro Ito, Atsushi Kawakami, Kiyosi Migita

**Affiliations:** ^1^Departments of General Internal Medicine and Rheumatology, Nagasaki Medical Center, Kubara 2-1001-1, Omura, Nagasaki 856-8562, Japan; ^2^Department of Pathology, Nagasaki Medical Center, Kubara 2-1001-1, Omura, Nagasaki 856-8562, Japan; ^3^Department of Ophthalmology, Nagasaki Medical Center, Kubara 2-1001-1, Omura, Nagasaki 856-8562, Japan; ^4^Department of Otolaryngology, Nagasaki Medical Center, Kubara 2-1001-1, Omura, Nagasaki 856-8562, Japan; ^5^Department of Rheumatology, Nagasaki University Hospital, Sakamoto 1-7-1, Nagasaki 852-8501, Japan

## Abstract

Granulomatosis with polyangiitis (GPA) sometimes involves the eye orbit; however, choroidal involvements in GPA had been rarely reported. We report a rare case presenting with a choroidal mass in an 83-year-old Japanese woman who presented with left eye pain. Diagnostic biopsy revealed necrotizing vasculitis with infiltrates of inflammatory cells. Diagnosis was localized granulomatosis with polyangiitis. Combined treatments with corticosteroid plus azathioprine resolved the choroidal mass region. Although treatment with corticosteroid and immunosuppressive agents improves the prognosis of the disease, ocular morbidity is still well recognized. Clinicians should consider a differential diagnosis of GPA in patients with inflammatory choroidal tumors.

## 1. Introduction

Granulomatosis with polyangiitis (GPA) is classically characterized as the triad of necrotizing granulomatosis lesions of the upper and lower respiratory tract, focal segmental glomerulonephritis, and necrotizing vasculitis [1]. The vasculitis preferentially affects small-fiber arterial vessels and, to a lesser extent, small and large vessels, causing arteritis [2]. Affected patients usually present with the disease in the upper respiratory tracts, lungs, and kidneys, although it has been reported to involve almost any organ [3]. GPA also can manifest as ophthalmological disease [4], although there have been few reports of choroidal involvement. Anti-neutrophil cytoplasmic antibody- (ANCA-) directed proteinase-3 (RR3-ANCA) is a diagnostic marker for GPA [5]. A definitive diagnosis of GPA, however, requires the presence of a histologically proved granulomatous inflammatory process, vasculitis, and giant cells [6]. We report the unusual clinical course of a patient with a choroidal tumor accompanied by an elevated serum myeloperoxidase- (MPO-) ANCA titer. The final diagnosis was identified as GPA when the pathological evaluation revealed the presence of necrotizing vasculitis and the serum MAO-ANCA assay was positive. We discuss here this unusual choroidal manifestation of GPA.

## 2. Case Presentation

An 83-year-old Japanese woman was referred to us with an 8-month history of left periorbital and left eye pain associated with headache migrating temporal region. The patient subsequently developed conjunctivitis of the left eye. The medical history included unstable angina 3 months previously that was treated with percutaneous coronary intervention. Upon admission, the patient had a temperature of 37.3°C, pulse 91/min, and blood pressure 90/63 mmHg. Her lungs were clear, and her abdomen was soft without hepatosplenomegaly. Neurological examinations revealed no nervous system involvements including cranial nerve palsy or peripheral neuropathy. The patient's pupils were both 3.0 mm, and motility was normal. Confrontational visual-field examinations were normal in both eyes. Intraocular pressures were 16 mmHg on the right and 17 mmHg on the left. Funduscopic examination revealed no abnormalities of the optic nerve or retinal vessels of both eyes.

Laboratory results are shown in Table 1. The patient's erythrocyte sedimentation rate was 86 mm/hr, and her serum C-reactive protein level was 9.73 mg/dL. The peripheral blood white blood cell count was 8400/*μ*L with normal renal function and urinalysis.

**Figure 1:**
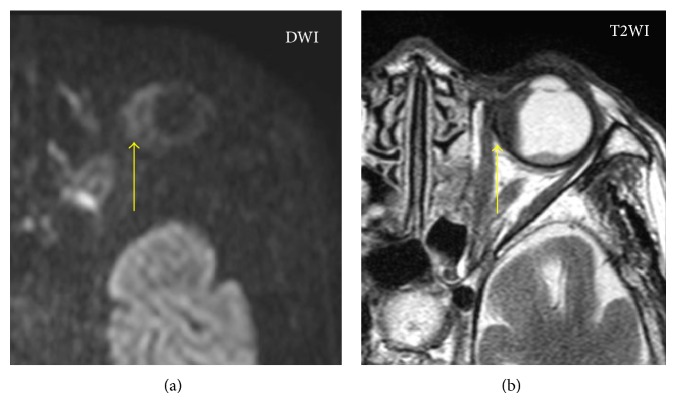
MRI images of the choroidal mass. Diffuse-weighted images (DWI) showed high signal intensity in left interior orbital lesion. T2-weighted image (T2WI) showed the choroidal tumor with low intensity of left eye.

**Figure 2:**
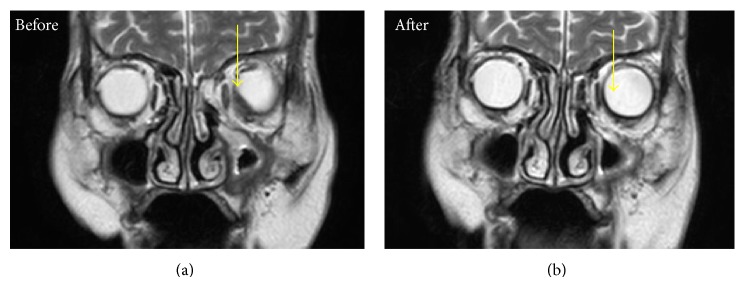
Coronal T2-weighted images showing a left choroidal tumor and space-occupying lesions in left paranasal sinus (arrow) before and 3 weeks after steroid treatment.

**Figure 3:**
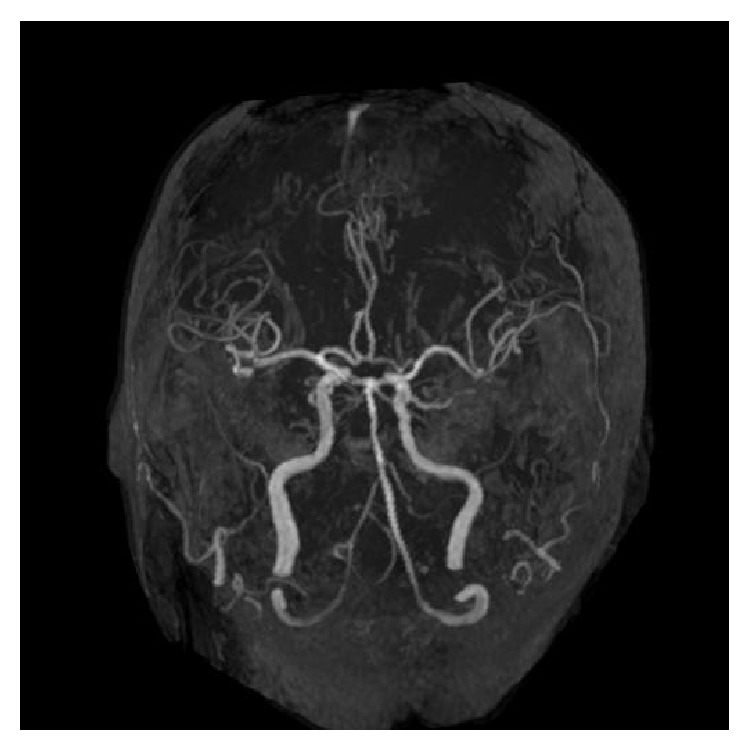
Magnetic resonance arteriography (MRA) before corticosteroid therapy shows no finding of cerebral vasculitis.

Enzyme-linked immunosorbent assays (ELISAs) showed positive results for MPO-ANCA (41.1 IU/mL, normal < 10 IU/mL) and negative results on an anti-proteinase-3- (PR3-) ANCA (3.5 IU/mL, normal < 10 IU/mL). Computed tomography (CT) of the chest and abdomen revealed no abnormal findings (data not shown). In magnetic resonance imaging (MRI) scans of the orbits, diffuse-weighted images (DWI) showed a mass lesion in the interior of left eye. T2-weighted image (T2WI) showed a choroidal tumor with low intensity in left eye (Figure 1). MRI also demonstrated space-occupying lesions in left paranasal sinus (Figure 2). Chest X-ray showed no significant finding and magnetic resonance arteriography (MRA) did not reveal the findings of cerebral vasculitis (Figure 3). There was no pulmonary or renal involvement.

**Figure 4:**
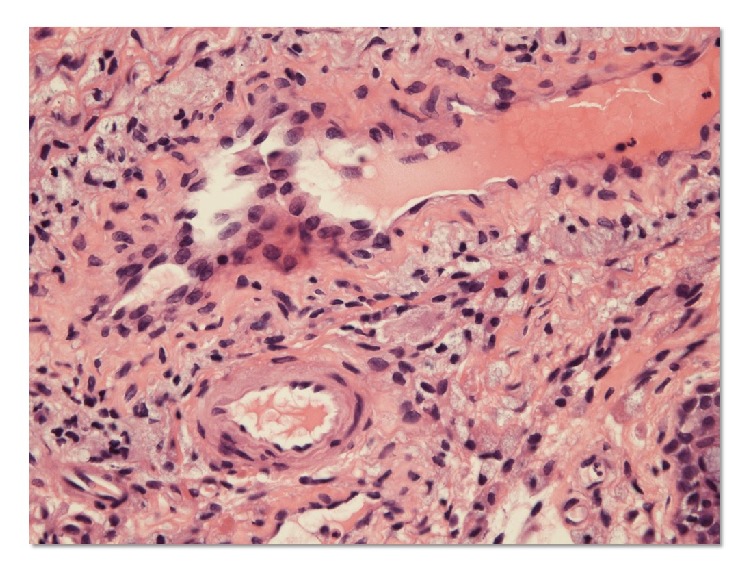
Histology of the left choroidal tumor showing vasculitis of a small-sized artery. Choroidal tumor biopsy specimens showing necrotizing vasculitis with aggregate of inflammatory cells (hematoxylin and eosin staining).

**Figure 5:**
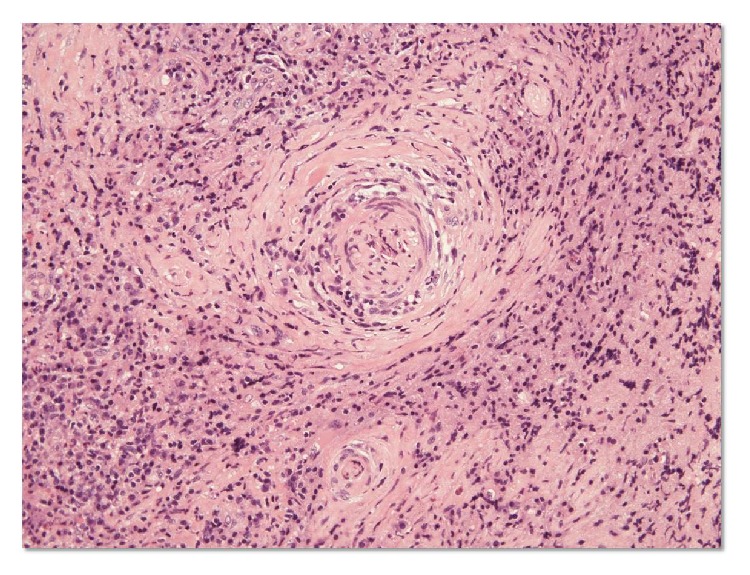
Histology of paranasal sinus biopsy specimens. Paranasal sinus biopsy specimens showing vasculitis with fibrinoid necrosis and small-size artery obliterated by concentric inflammatory cells with granulomatous inflammation (hematoxylin and eosin staining).

We performed diagnostic biopsy of the choroidal mass. Histopathological evaluation of the specimen revealed granulomatous choroiditis with necrotizing vasculitis with infiltrates of inflammatory cells (Figure 4). Also, surgical biopsy specimens of left paranasal sinus showed vasculitis of small-sized vessels with fibrinoid necrosis (Figure 5). According to the international Chapel Hill Consensus Conference (CHCC) nomenclature for vasculitis syndrome [7], the patient had small-vessel vasculitis and the diagnosis of limited type of GPA was made based on the serological (presence of MPPO-ANCA) and histological evidence of vasculitis and granulators inflammation in choroidal and sinus tissues, without the involvement of respiratory tracts and kidney. Other small-vessel vasculitides, such as microscopic polyangiitis (MPA) or eosinophilic granulomatosis with polyangiitis (EGPA), were ruled out from these clinical findings.

**Figure 6:**
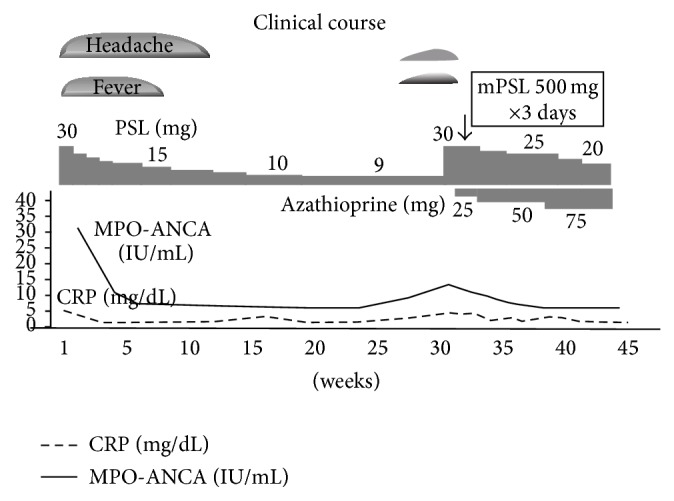
Clinical course of the present case.

We treated the patient with oral prednisolone (30 mg/day). The symptoms rapidly resolved. MRI findings indicated that the choroidal tumor was also rapidly resolving (Figure 2). Two months later, she was symptom-free with normalized MPO-ANCA levels on orally prednisolone 10 mg daily. However, 3 months later, she experienced a relapse of fever with elevated levels of CRP and MPO-ANCA. She was treated with methylprednisolone pulse therapy (500 mg, 3 successive days) and we increased her prednisolone to 30 mg a day. She was then started with azathioprine (50 mg daily) and her prednisolone was eventually tapered down to 20 mg/day over three months. She continues to be doing well with no further relapse (Figure 6).

## 3. Discussion

We report the case of a choroidal tumor in a patient with GPA. Choroidal tumors are not uncommon, but the development of a choroidal tumor caused by GPA is rare. To our knowledge, no choroidal tumor as a direct consequence of GPA has been described, although retrobulbar granuloma is one of the serious complications of GPA [8]. It is often resistant to conventional therapy, and the outcome usually includes visual loss and orbital deformity [9]. Until recently, such patients had undergone surgical resection [9].

In our case, histological examination showed a chronic necrotizing granulomatous inflammation localized in the choroidal tissues, thus creating the picture of a solid choroidal tumor. These clinical and histological findings seem to indicate that choroidal granuloma may be another ocular manifestation of GPA. Although GPA can affect any systemic organ, ophthalmological manifestations occur in 28~77% of GPA patients [10]. The most common manifestation of GPA is orbital disease (15%), followed by scleral, episcleral (3.5%), corneal (8%), and nasolacrimal (7%) abnormalities [11]. The presence of a solid choroidal tumor in patients with GPA is unusual, although choroidal involvement in GPA may manifest as uveitis, retinal vasculitis, choroidal arterial occlusion, or choriocapillaritis. GPA is a systemic inflammatory disease whose histological features often include necrosis, granuloma formation, and vasculitis of small to medium-sized vessels [12]. ANCAs are present in about 80–90% of patients and appear to play a role in the pathogenesis of GPA [5]. RR3-ANCA is a diagnostic marker of GPA [5]. A definitive diagnosis of GPA, however, requires the presence of a histologically proved granulomatous inflammatory process, vasculitis, and the presence of giant cells [6]. GPA was diagnosed in our patient based on the pathological findings. The presence of MPO-ANCA in this patient was somewhat atypical. This finding was similar to that in the GPA case series reported by Chen in which the PR3-ANCA assay was negative and the assay for MPO-ANCA was positive [13]. Therefore, the presence of MPO-ANCA in these patients is not inconsistent with GPA.

The gold standard treatment for GPA combines glucocorticoids (GCs) and cyclophosphamide (CYP) [14]. More severe ocular disease is often not responsive to GCs alone. The use of oral GCs alone achieved good control in a limited number of patients exhibiting serious deterioration [4]. The most dramatic benefits have occurred with the use of GC and CYP. It is therefore believed that the treatment of choice for significant GPA with an ocular component remains the GC-CYP combination [4]. In ANCA-associated vasculitis, glucocorticoid tapering protocol, including the use of less toxic immunosuppressants such as azathioprine in remission-maintenance therapy, has been proposed [15]. The Japanese patients with MPO-ANCA-associated vasculitis (JMAAV) trial revealed the usefulness of severity-based treatment, in which low dose corticosteroid and, if necessary, cyclophosphamide or azathioprine were recommended in patients with mild form [16]. The present case was successfully treated by GCs plus azathioprine against clinical manifestations of GPA. Thus, the less toxic regimens may be considered initially in cases of less aggressive, limited disease, especially in elderly patients with GPA.

In summary, we report a case of choroidal tumor in a patient with granulomatous choroiditis secondary to GPA. GPA is a rare entity that can affect almost any organ system. To our knowledge, no choroidal tumor has been previously described as a direct consequence of GPA. Although ophthalmic involvement is relatively common during the course of the disease, choroidal involvement is rare. It is important, however, to consider GPA in the differential diagnosis of a choroidal tumor. Early diagnosis and an appropriate interdisciplinary approach to management are required to decrease morbidity in patients with GPA-mediated inflammatory ocular disease.

## Figures and Tables

**Table 1:** Laboratory findings.

*Peripheral blood *	
Red blood cells	337 × 10^4^/*μ*L
Hemoglobin	10.4 g/dL
White blood cells	8400/*μ*L
Neutrophil	83.2%
Monocyte	5.5%
Lymphocyte	10.0%
Eosinophil	0.9%
Basophil	0.5%
Platelet	30.4 × 10^4^/*μ*L
*Blood chemistry *	
Total protein	7.6 g/dL
Albumin	2.8 g/dL
Total bilirubin	0.5 mg/dL
Glutamic-oxaloacetic transaminase	19 IU/L (7–33)
Glutamic-pyruvic transaminase	14 IU/L (5–30)
Lactate dehydrogenase	199 IU/L (260–480)
Alkaline phosphatase	261 IU/L (80–250)
Creatinine kinase	39 IU/L (60–160)
Total cholesterol	171 mg/dL
Blood urea nitrogen	15.0 mg/dL
Creatinine	0.4 mg/dL
Na	132 mEq/L
K	4.1 mEq/L
Cl	100 mEq/L
Ca	8.7 mg/dL
*Serological tests *	
C-reactive protein	9.37 mg/dL (<0.30)
Erythrocyte sedimentation rate	86 mm/hr
IgG	2250 mg/dL (900–2000)
IgA	458 mg/dL
IgM	131 mg/dL
C3	155 mg/dL (86–160)
C4	50 mg/dL (17–45)
Rheumatoid factor	24 (U/mL) (<17)
Antinuclear Ab	<×40 (Speckled)
PR-3-ANCA	<1.0 U/mL (<3.5)
MPO-ANCA	41.1 U/mL (<3.5)
Anti-CCP Ab	<0.6 U/mL (<4.5)
*Virological test *	
HCV-Ab	(−)
HBsAg	(−)
*Urinalysis *	
Protein	(−)
Sugar	(−)
Occult blood	(−)
Sediment	np

HBsAg: hepatitis B surface antigen; Anti-CCP Ab: anti-cyclic citrullinated peptide antibody; HCV: hepatic C virus; MPO-ANCA: myeloperoxidase anti-neutrophil cytoplasmic antibody; PR-3-ANCA: proteinase 3-anti-neutrophil cytoplasmic antibody.
